# Postoperative rehabilitation after transtibial pullout repair of medial meniscus posterior root tears: A systematic review and meta‐analysis

**DOI:** 10.1002/ksa.70491

**Published:** 2026-06-16

**Authors:** Mutaz Mohamed Tageldein, Yasmin Alhamdah, Marc Daniel Bouchard, Prushoth Vivekanantha, Ben Murphy, Yuichi Hoshino, Bryson Lesniak, Darren de SA

**Affiliations:** ^1^ Michael G. DeGroote School of Medicine McMaster University Hamilton Ontario Canada; ^2^ School of Medicine St. George's University West Indies Grenada; ^3^ Division of Orthopaedic Surgery, Department of Surgery McMaster University Hamilton Ontario Canada; ^4^ Hamilton Orthopaedic Institute Stoney Creek Ontario Canada; ^5^ Department of Orthopaedic Surgery Kobe University Graduate School of Medicine Kobe Japan; ^6^ Division of Sports Medicine, Department of Orthopaedic Surgery University of Pittsburgh Medical Center Pittsburgh Pennsylvania USA

**Keywords:** medial meniscal posterior root tear (MMPRT) repair, patient‐reported outcomes, rehabilitation protocols, transtibial pullout repair

## Abstract

**Purpose:**

To characterise rehabilitation protocols after transtibial pullout repair for medial meniscus posterior root tears (MMPRTs) and evaluate whether study‐level rehabilitation parameters were associated with longitudinal changes in patient‐reported outcome measures (PROMs).

**Methods:**

A systematic database search was conducted from inception to 2 June 2025, and updated on 2 February 2026. Study‐level data were extracted, including rehabilitation protocols and PROMs. For each study, mean change scores were calculated as the difference between post‐operative follow‐up and preoperative baseline PROM values. When not reported, the standard deviation of the change score was imputed from baseline and follow‐up standard deviations assuming a pre‐post correlation (*r*) of 0.5; sensitivity analyses used *r* = 0.3 and *r* = 0.7. Random‐effects meta‐analysis with Hartung–Knapp adjustment determined overall PROM improvement, and rehabilitation variable effects were tested through study‐level meta‐regression.

**Results:**

Overall, 31 studies were included, with 17 in the meta‐analysis. Pooled mean change scores demonstrated substantial post‐operative improvements in International Knee Documentation Committee (IKDC) (28.5 [95% confidence interval [CI] 23.8–33.2]), Knee injury and Osteoarthritis Outcome Score Quality of Life (KOOS QoL) (33.9 [95% CI 22.2–45.6]), KOOS Sport/Rec (33.1 [95% CI 26.0–40.3]), and visual analog scale (VAS) pain (−34.6 [95% CI −40.1 to −29.1]) scores. Pooled estimates were stable using alternative assumptions for change‐score variance. Comparing USA and Asian cohorts, bracing duration differed significantly, with USA cohorts more commonly using post‐operative bracing for ≥6 weeks; other evaluated rehabilitation variables did not differ significantly. No detectable associations were identified in exploratory study‐level meta‐regression analyses, although between‐study heterogeneity was substantial.

**Conclusion:**

Rehabilitation protocols after transtibial pullout repair for MMPRTs are heterogeneous. Patients demonstrated substantial longitudinal PROM improvements; however, no detectable associations were identified between evaluated rehabilitation protocol variables and IKDC improvement. These findings are limited by observational study design, high heterogeneity, ecological bias and insufficient power.

**Level of Evidence:**

Level IV.

**Abbreviations**
ACL: anterior cruciate ligament
BMI: body mass index
CENTRAL: Cochrane Central Register of Controlled Trials
CI: confidence interval
CINAHL: Cumulative Index to Nursing and Allied Health Literature
CS: case series
I^2^
: I‐squared
IKDC: International Knee Documentation Committee
KOOS: Knee Injury and Osteoarthritis Outcome Score
MCID: minimal clinically important Difference
MeSH: Medical Subject Heading
MINORS: Methodological Index for Non‐Randomised Studies
MMPRT: medial meniscus posterior root tear
N: no
NA: not applicable
NR: not reported
NWB: non‐weightbearing
OR: odds ratio
PC: Prospective Cohort Study
PRISMA: Preferred Reporting Items for Systematic Reviews and Meta‐Analyses
PROMs: Patient‐Reported Outcome Measures
PWB: partial weight‐bearing
QoL: quality of life
r: pre‐post correlation coefficient
RC: Retrospective Cohort Study
RCT: randomised controlled trial
RoB 2: Risk of Bias 2
ROM: range of motion
SD: standard deviation
USA: United States of America
VAS: Visual Analog Scale
WBAT: weight‐bearing as tolerated
Y: yes

## INTRODUCTION

Medial meniscus posterior root tears (MMPRTs) are avulsions or radial tears within one centimetre of the tibial insertion of the posterior horn. These tears interrupt the circumferential ‘hoop’ tension of the meniscus and biomechanically approximate the effects of a subtotal meniscectomy, leading to rapid compartment overload and accelerated medial tibiofemoral osteoarthritis [1, 11, 33]. Recent studies report that root tears represent roughly 15%–20% of all meniscus tears and the medial posterior root is the most frequently torn. This is especially true in middle‐aged or older patients, women, those with a higher body mass index (BMI) and knees with a varus alignment [10, 11, 18, 30]. MMPRTs often coexist with meniscal extrusion and early cartilage changes, thus accelerating progression to symptomatic osteoarthritis if the root is not restored [30, 33].

Treatment strategies for MMPRTs have evolved from partial meniscectomy or nonoperative care towards meniscal root restoration to re‐establish meniscal function. Several arthroscopic techniques have been described, including transtibial pullout repair, suture‐anchor (all‐inside) repair, and hybrid techniques. Among these, the transtibial pullout repair remains one of the most commonly used techniques because it is reproducible and adaptable across tear morphologies [13, 17, 34]. However, outcomes appear to be influenced by age, BMI, varus alignment, baseline degenerative changes, meniscal extrusion, chronicity and surgical technique, suggesting that surgical repair alone may not determine clinical success [13, 17, 34].

Post‐operative rehabilitation protocols after MMPRT repair are less standardised than surgical techniques and remain highly variable. Protocols often differ across key early‐phase domains: immobilisation/bracing, weight‐bearing progression, and range‐of‐motion (ROM) restrictions. Rehabilitation protocols may also vary across geographic regions because of differences in surgeon training patterns and institutional norms for post‐operative care. Contemporary protocols range from 6 weeks of non‐weightbearing or toe‐touch weight‐bearing in a brace locked in extension to accelerated programs that permit immediate weight‐bearing and early ROM, with comparable short‐term clinical outcomes reported [8, 21, 47]. Studies often report good clinical outcomes without clearly linking results to specific rehabilitation parameters used, making cross‐study interpretation difficult. Although most MMPRT repair studies report on PROMs, and often show improvements after repair, these improvements are not analysed against rehabilitation parameters. Thus, it is unknown whether specific protocols or protocol parameters are associated with better outcomes.

Recent European Society for Sports Traumatology and Arthroscopy, American Orthopedic Society for Sports Medicine and American Academy of Sports Physical Therapy (ESSKA‐AOSSM‐AASPT) consensus recommendations provide contemporary guidance for rehabilitation after meniscus surgery, including combined time‐ and criterion‐based progression after meniscal repair and more protective restrictions for root repairs [37]. Specifically, the consensus recommends no weight‐bearing for six weeks and ROM restriction for 0°–90° for the early post‐operative period after root repair [37]. However, MMPRT‐specific comparative evidence remains limited, and it is unclear whether the rehabilitation parameters reported in clinical studies are associated with differences in patient‐reported outcomes. Accordingly, the purpose of this systematic review and meta‐analysis was to characterise reported post‐operative rehabilitation protocols after transtibial pullout repair for MMPRTs and evaluate whether study‐level rehabilitation parameters were associated with longitudinal PROM improvements. We hypothesised that reported rehabilitation protocols would be heterogeneous and that more conservative rehabilitation parameters would be associated with greater improvements in PROMs compared to more aggressive rehabilitation parameters.

## METHODS

This systematic review and meta‐analysis adhered to the Preferred Reporting Items for Systematic Reviews and Meta‐Analyses guidelines [35].

### Search strategy

Medline, Embase, Cochrane Central Register of Controlled Trials (CENTRAL), Cumulative Index to Nursing and Allied Health Literature (CINAHL), and SPORTDiscus databases were systematically searched from inception until 2 June 2025. Our search strategy utilised Medical Subject Heading (MeSH) terms and keywords, including (“menisc*” OR “meniscal”) AND (“posterior horn” OR “posterior root”) AND (“tear” OR “injury” OR “avulsion” OR “lesion”) AND (“repair” OR “sutur*” OR “fixat*” OR “reattach” OR “pull‐out” OR “pullout” OR “transtibial” OR “tunnel*” OR “anchor” OR “reconstruction” OR “centralisation”) with Boolean operators for precise filtering. The full search strategy can be found in Supporting Information: Appendix 1.

Supplemental searching was conducted by team members using citation search via Google Scholar, PubMed and the reference lists of included studies to identify any additional articles missed in the initial yield. In addition, continued literature surveillance was conducted until 2 February 2026, to identify newly published eligible studies.

### Study selection

Title, abstract and full‐text screening of identified articles for inclusion were performed independently by two reviewers (MMT and YA) using Covidence software (Veritas Health Innovation, Melbourne, Australia). Conflicts at all stages were resolved through discussion with a senior author (MDB). Eligible studies included randomised controlled trials, cohort studies, case series (≥10 patients) or case control studies of adult (≥18 years old) patients who have undergone transtibial pullout repair of medial meniscus posterior root tears. Studies with multiple techniques were eligible for inclusion if data for the transtibial pullout arm was extractable. Additionally, eligible studies had to describe at least one element of post‐operative rehabilitation (brace, weight‐bearing, range of motion [ROM], axial loading, etc.). No restrictions on the year of publication were made. Non‐English studies, conference abstracts and studies without an available full text were excluded. Additional exclusions included paediatric, animal or cadaveric studies, and studies involving major concomitant procedures that substantially alter post‐operative rehabilitation (e.g., anterior cruciate ligament reconstruction and high tibial osteotomy). Studies that performed adjunctive meniscal centralisation were retained if the cohort otherwise underwent transtibial pullout repair for MMPRT, as centralisation was considered a surgical technique adjunct rather than a separate rehabilitation‐altering concomitant procedure.

### Data extraction and abstraction

Data from included studies were independently extracted by two reviewers (MMT and YA) using a standardised collection sheet on Microsoft Excel version 16.90 (Microsoft Corporation, Redmond, WA, USA). Extracted data included study characteristics (author, country, year of publication, study design, sample size and follow‐up duration), patient characteristics (age and sex), rehabilitation protocol details (bracing, weight‐bearing, ROM, axial loading and return to activity/sport), failure/re‐tear rates, complications and PROMs (including but not limited to Visual Analog Scale [VAS] Pain, International Knee Documentation Committee [IKDC] and Knee Injury and Osteoarthritis Outcome Score [KOOS]).

### Study quality assessment

For randomised controlled trials (RCTs), the Cochrane Risk of Bias 2.0 (RoB 2) tool was applied to examine five key domains: adequacy of the randomisation process, fidelity to intended interventions, completeness of outcome data, reliability of outcome measurement and risk of selective reporting. Each domain was rated as “low risk,” “some concerns,” or “high risk,” with the overall rating based on the domain with the highest risk [41].

For prospective cohort studies, retrospective cohort studies and case series, the Methodological Index for Non‐Randomised Studies (MINORS) score was used to assess study quality [40]. For non‐comparative studies, the MINORS score ranges from 0 to 16, where ≥ 13 is considered high quality, 8–12 fair, 5–7 low and 0–4 very low quality. For comparative studies, the MINORS score ranges from 0 to 24, where ≥ 20 is considered high quality, 12–19 fair, 7–11 low and 0–6 very low quality. All included studies were assessed by two reviewers (MMT and YA) who independently completed the risk of bias assessment; a senior author (MDB) helped resolve any disagreement.

### Statistical analysis

Statistical analyses were performed using R (version 4.5.1). For PROMs with sufficient extractable data, mean change was calculated as post‐operative follow‐up minus preoperative baseline. Because included studies did not compare rehabilitation strategies within randomised or controlled rehabilitation groups, pooled PROM estimates were interpreted as longitudinal post‐operative changes rather than causal treatment effects attributable to rehabilitation or surgery alone.

When change‐score standard deviations were not reported, they were derived from preoperative and post‐operative standard deviations assuming a pre‐post correlation of *r* = 0.5; sensitivity analyses were performed using *r* = 0.3 and 0.7 [14]. Random‐effects meta‐analysis with Hartung–Knapp adjustment was used to estimate pooled mean change and 95% confidence intervals. Heterogeneity was assessed using *I* ^2^. Publication bias/small‐study effects were assessed for IKDC outcomes using visual inspection of funnel plot asymmetry and Egger's regression test.

Regional differences in rehabilitation protocols were assessed using Fisher's exact test, excluding studies where the variable was not reported. Exploratory study‐level meta‐regression was performed using rehabilitation variables as moderators. Rehabilitation variables were analysed using a priori thresholds reflecting commonly reported protocol distinctions and contemporary consensus guidance for root repairs as follows: brace use duration (<6 vs. ≥6 weeks), locking in extension (yes vs. no), duration of locking in extension (<2 vs. ≥2 weeks), immediate post‐operative weight‐bearing status (non‐weightbearing [NWB] vs. partial weight‐bearing [PWB, includes toe‐touch weight‐bearing]), time to weight‐bearing as tolerated (WBAT, ≤6 vs. >6 weeks) and immediate ROM allowed (yes vs. no) [30]. Binary moderator analyses were performed only when at least three studies were available in each subgroup. We acknowledge that three studies per subgroup may still be insufficient for reliable moderator analyses; however, given the expected limited number of studies available for quantitative synthesis, this threshold was selected a priori as a minimally acceptable criterion for exploratory analysis. These analyses were considered exploratory and interpreted cautiously because of sparse study counts, ecological bias, residual confounding, high heterogeneity and multiple testing. All tests were two‐sided, and a *p*‐value < 0.05 was considered statistically significant.

## RESULTS

### Search results

**Figure 1:**
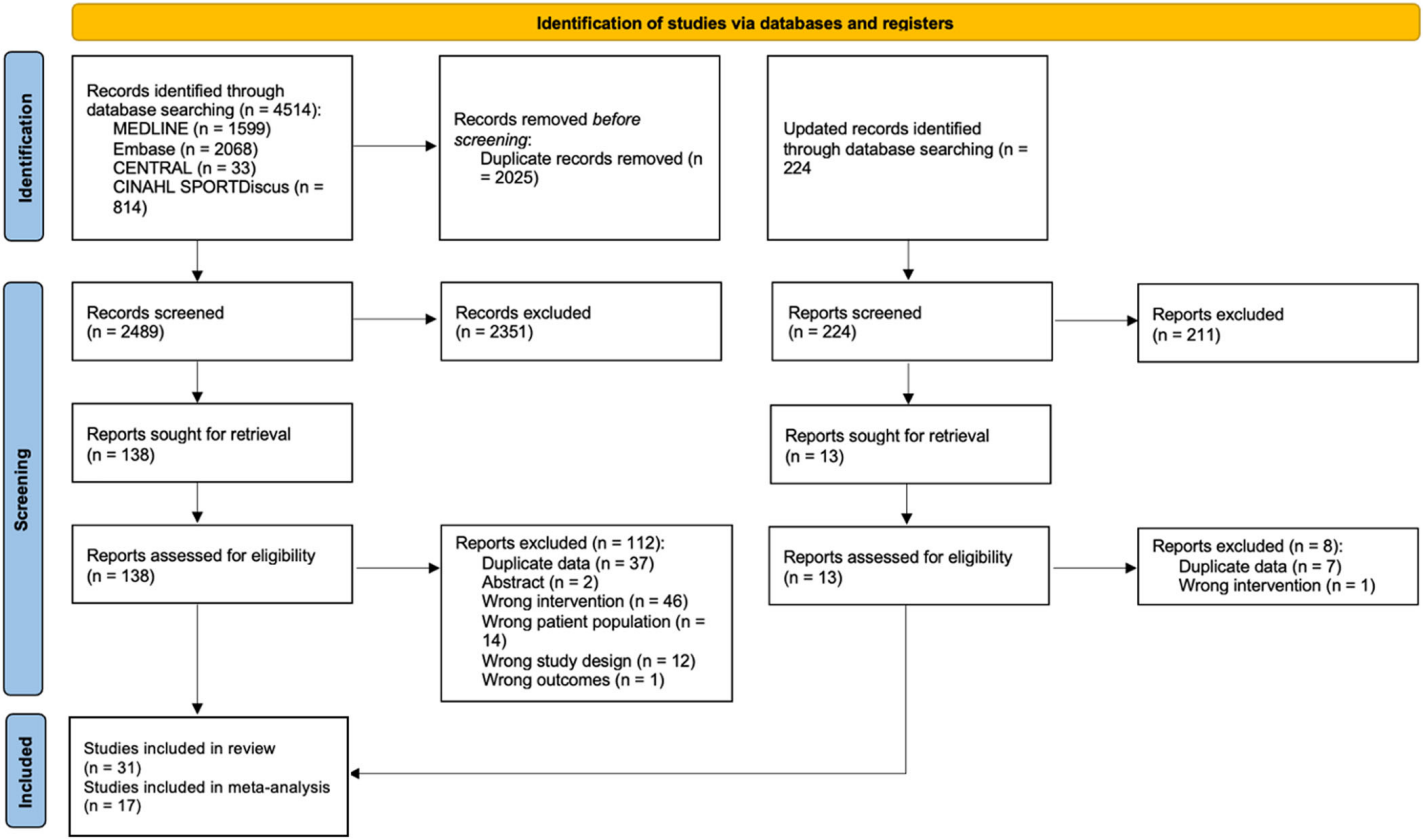
PRISMA flow diagram.

A total of 4514 articles were identified from the search of the databases. Following duplicate removal and screening of titles and abstracts, 138 full‐text articles were assessed for eligibility. After an updated search, 13 additional full‐text reports were assessed for eligibility. In total, 31 articles were included for qualitative analysis [1, 2, 3, 4, 5, 6, 7, 9, 12, 15, 16, 20, 22, 23, 25, 26, 27, 28, 31, 32, 33, 39, 42, 43, 44, 45, 46, 48, 49, 50, 51], and 17 studies were included in the quantitative analysis (Figure 1) [1, 12, 15, 16, 20, 22, 23, 25, 26, 27, 28, 31, 32, 43, 44, 48, 50].

### Study characteristics and rehabilitation protocol variability

Demographic data and study characteristics are shown in Table 1 and Supporting Information: Appendix 2. This review included 31 studies and 1287 patients undergoing transtibial pullout repair for MMPRT. Most studies were retrospective cohorts; four prospective cohort studies, two randomised controlled trials and two case series of 21 and 14 patients were also included. The median sample size was 33.0 patients (range 12–179), reflecting predominantly single‐center studies.

**Table 1:** Demographic data and study characteristics of included studies.

Study	Country	Study design	Sample size (*n*)	Age (years)	Female sex: *n* (%)	Follow‐up, years
Allende et al. [1]	USA	RC	70	55.2 ± 10.7	51 (72.9)	2.9 ± 1.0
Bagherifard et al. [2]	Iran	RC	16	50.9 ± 11.0	14 (87.5)	1.6 ± 1.2
Chung et al. [7]	Korea	RC	34	58.2 ± 6.1	30 (88.2)	1.0 ± 0.1
Kawada et al. [20]	Japan	RC	179	65.5 ± 8.3	147 (82.1)	2.0^a^
Kizilay and Çamurcu [22]	Turkey	RC	41	49.0 ± 12.0	32 (78.0)	2.7 ± 0.9
Li et al. [28]	China	RCT	20	37.7 ± 1.2	11 (55.0)	NR
Nistor et al. [33]	Romania	CS	14	59.0 ± 13.0	9 (64.3)	1.5 ± 0.7
Tollefson et al. [45]	USA	RCT	12	56.1 ± 7.1	9 (75.0)	0.6 ± 0.2
Herber et al. [12]	USA	RC	97	56.1 ± 9.9	74 (76.3)	2.0 ± NR
Krych et al. [23]	USA	PC	25	50.1 ± 11.3	19 (76.0)	2.0 ± 0.6
Moore et al. [32]	USA	RC	17	47.2 ± 12.2	11 (64.7)	9.2 ± 0.6
Tamura et al. [44]	Japan	RC	48	65.0 ± 7.9	39 (81.3)	2.0 ± NR
Yan et al. [48]	China	RC	23	59.8 ± 7.9	12 (52.2)	1.1 ± NR
Zhou et al. [51]	China	RC	87	56.4 ± 5.9	53 (60.9)	2.2 ± 0.2
Chen et al. [5]	Taiwan	RC	48	58.6 ± 9.9	32 (66.7)	0.9 ± 0.7
Takase et al. [43]	Japan	PC	19	64.1 ± 9.9	13 (68.4)	1.0 ± NR
Yoon et al. [50]	Korea	RC	16	54.3 ± 9.6	14 (87.5)	2.2 ± 0.3
Dzidzishvili et al. [9]	Spain	RC	30	52.2 ± NR	NR	2.3 ± 0.4
Li et al. [27]	China	PC	35	36.1 ± 10.9	15 (42.9)	2.2 ± 0.2
Tahami et al. [42]	Iran	RC	43	53.2 ± 8.1	39 (90.7)	NR
Hopkins and Lowrie [16]	Australia	PC	33	56.8 ± 9.7	23 (70.0)	3.3 ± 1.3
Moon et al. [31]	Korea	RC	73	55.2 ± 8.4	58 (79.5)	NR
Bernard et al. [3]	USA	RC	15	46.1 ± NR	10 (66.7)	3.3 ± NR
Hiranaka et al. [15]	Japan	RC	68	64.4 ± 7.9	53 (77.9)	1.0 ± NR
Lee et al. [25]	Korea	RC	47	56.2 ± 6.5	44 (93.6)	2.2 ± 0.4
Ulku et al. [46]	Turkey	RC	41	52.9 ± 3.8	36 (87.8)	3.7 ± NR
Yanagisawa et al. [49]	Japan	RC	30	60.7 ± 8.3	25 (83.3)	1.0 ± NR
Brophy et al. [4]	USA	RC	22	50.4 ± 11.0	14 (64.0)	2.4 ± 0.3
Cho and Song [6]	Korea	RC	13	50.3 ± NR	12 (92.3)	0.6 ± NR
Lee et al. [26]	Korea	RC	50	56.1 ± 8.7	46 (92.0)	2.1 ± 0.5
Seo et al. [39]	Korea	CS	21	52.3 ± NR	17 (81.0)	1.1 ± NR
**Median (range)**			**33.0 (12–179)**	**56.1**	**‐**	**2.0 (0.6–9.2)**
**Total**			**1287**	**‐**	**962/1257 (76.5)**	**‐**

*Note*: Values are presented as mean ± SD unless otherwise stated.

Abbreviations: CS, case series; NR, not reported; PC, prospective cohort study; RC, retrospective cohort study.

^a^ Meniscal extrusion and second‐look arthroscopy mean follow‐up was 1.0 years.

Of the included studies that report on brace duration, the median was 4.0 weeks of bracing (range 0–10) with 16 (57.1%) of the reporting studies locking in extension postoperatively (Table 2). The median duration of being locked in extension was 2.0 weeks (range 1–10) with 15 (50.0%) protocols allowing ROM immediately postoperatively. Most rehabilitation protocols (21, 67.7%) included non‐weightbearing immediately after surgery with a median of 6 weeks to weight‐bearing as tolerated (range 4–12).

**Table 2:** Rehabilitation protocol details of included studies.

Study	Brace use duration (weeks)	Locked in extension (Y/N)	Locked in extension duration (weeks)	Immediate post‐operative weight‐bearing status (NWB/PWB)	Time to WBAT (weeks)	Immediate ROM allowed (Y/N)
Allende et al. [1]	6	N	NA	NWB	6	Y
Bagherifard et al. [2]	6	Y	2	NWB	12	N
Chung et al. [7]	3	Y	3	PWB	6	N
Kawada et al. [20]	1	Y	1	NWB	4	N
Kizilay and Çamurcu [22]	NR	Y	NR	NWB	NR	Y
Li et al. [28]	2	N	NA	NWB	7	Y
Nistor et al. [33]	0	N	NA	NWB	4	Y
Tollefson et al. [45]	NR	NR	NR	NWB	6	Y
Herber et al. [12]	6	Y	1.4	PWB	6	N
Krych et al. [23]	4	N	NA	NWB	4	Y
Moore et al. [32]	6	Y	6	NWB	12	N
Tamura et al. [44]	2	Y	2	NWB	6	N
Yan et al. [48]	4	Y	1	NWB	6	N
Zhou et al. [51]	6	N	NA	NWB	6	Y
Chen et al. [5]	NR	NR	NR	NWB	NR	NR
Takase et al. [43]	4	Y	4	NWB	4	N
Yoon et al. [50]	6	Y	6	PWB	6	N
Dzidzishvili et al. [9]	NR	N	NA	NWB	6	Y
Li et al. [27]	NR	N	NA	NWB	6	Y
Tahami et al. [42]	2	Y	2	PWB	6	N
Hopkins and Lowrie [16]	6	N	NA	NWB	NR	Y
Moon et al. [31]	10	Y	10	PWB	10	N
Bernard et al. [3]	6	N	NA	PWB	6	Y
Hiranaka et al. [15]	2	N	NA	NWB	6	Y
Lee et al. [25]	NR	NR	NR	PWB	6	N
Ulku et al. [46]	5	Y	5	PWB	5	Y
Yanagisawa et al. [49]	4	N	NA	NWB	4	Y
Brophy et al. [4]	10	Y	10	NWB	NR	N
Cho and Song [6]	NR	Y	2	NWB	8	N
Lee et al. [26]	NR	N	NA	PWB	6	Y
Seo et al. [39]	2	Y	2	PWB	6	N
Median (range)	4.0 (0–10)	NA	2.0 (1–10)	NA	6 (4–12)	NA

Abbreviations: NA, not applicable; NR, not reported; NWB, non‐weightbearing; PWB, partial weight‐bearing; ROM, range of motion; WBAT, weight‐bearing as tolerated.

### Study quality

Risk of bias was assessed using the MINORS instrument for non‐randomised designs and the RoB 2 tool for randomised trials (Supporting Information: Appendices 3–4).

Across 29 non‐randomised studies, 14 studies were non‐comparative and were therefore scored out of 16; the median score was 9.5 (range 8–15), representing fair methodological quality. Fifteen studies were comparative and were scored out of 24, with a median score of 15 (range 10–18), representing fair methodological quality as well. Most studies consistently achieved high scores for having a clearly stated aim and using endpoints aligned with the study objective. In contrast, common methodological limitations included limited prospective elements such as study size calculations and data collection, loss to follow‐up greater than 5%, and, among comparative cohorts, variable performance on key comparative aspects. These patterns indicate that the evidence base is predominantly observational, with moderate methodological constraints that could introduce bias and imprecision.

Two randomised trials were assessed with RoB 2. One trial was judged high risk of bias overall, driven by a high‐risk rating in Domain 4 (measurement of the outcome) alongside “some concerns” in multiple additional domains. The second trial was rated as having some concerns overall, reflecting residual concerns across several domains despite a low‐risk rating for the randomisation process (Domain 1). Overall, the randomised evidence was limited in quantity and did not uniformly meet low‐risk standards across all bias domains.

### Variability in rehabilitation protocols by region

Rehabilitation protocols were reported across seven North American cohorts (all from the United States [USA]), 21 Asian cohorts, two European cohorts, and one Australian cohort. Variability was only compared between USA and Asian studies as other regions did not have sufficient study counts. Regional variability was compared between USA and Asian studies using Fisher's exact test, excluding non‐reported variables, as these regions comprised the majority of included studies.

The protocols differed across regions, with the most pronounced difference observed for post‐operative bracing duration (Table 3). Most USA cohorts that reported bracing duration maintained post‐operative bracing for at least 6 weeks (5/6, 83.3%). In contrast, most Asian cohorts that reported bracing duration discontinued bracing before 6 weeks (11/15, 73.3%), while 4/15 (26.7%) used bracing for ≥6 weeks. This difference was statistically significant (OR 0.07, *p* = 0.046), indicating that USA cohorts were more likely to use prolonged post‐operative bracing compared with Asian cohorts.

**Table 3:** Rehabilitation protocol variability by region.

Region	Number of studies		
Bracing duration			
	<6 weeks	≥6 weeks	Not reported
USA	1	5	1
Asia	11	4	6
Fisher's exact OR	0.073	*p*‐value	0.046
Locked in extension			
	N	Y	Not reported
USA	3	3	1
Asia	6	13	2
Fisher's exact OR	2.167	*p*‐value	0.63
Locked in extension duration			
	<2 weeks	≥2 weeks	Not reported
USA	1	2	4
Asia	2	10	9
Fisher's exact OR	2.500	*p*‐value	0.51
Immediate post‐operative weight‐bearing status			
	NWB	PWB	Not reported
USA	5	2	0
Asia	13	8	0
Fisher's exact OR	1.538	*p*‐value	1.00
Time to weight‐bearing as tolerated			
	≤6 weeks	>6 weeks	Not reported
USA	5	1	1
Asia	15	4	2
Fisher's exact OR	1.333	*p*‐value	1.00
ROM immediately postoperatively			
	N	Y	Not reported
USA	3	4	0
Asia	12	8	1
Fisher's exact OR	0.500	*p*‐value	0.66

Abbreviations: N, no; NWB, non‐weightbearing; OR, odds ratio; PWB, partial weight‐bearing; Y, yes.

Immobilising the knee in extension was used in half of the reporting US cohorts (3/6, 50.0%), of which two‐thirds kept the knee locked in extension for at least 2 weeks (2/3, 66.7%). Among Asian studies, 13/19 (68.4%) utilised this technique, with 10/12 (83.3%) keeping the knee in this position for at least 2 weeks. There was no evidence of a statistically significant regional difference in these variables.

Weight‐bearing progression was consistently reported and broadly similar across regions. Keeping the operative leg non‐weightbearing was common in both regions (USA: 5/7, 71.4%; Asia: 13/21, 61.9%) with most cohorts achieving weight‐bearing as tolerated within 6 weeks (USA: 5/6, 83.3%; Asia: 15/19, 78.9%).

Immediate post‐operative ROM was initiated in 4/7 US cohorts (57.1%) and 8/20 Asian cohorts (40.0%), without evidence of a regional difference (OR 0.50, *p* = 0.66). Overall, regional variability was most evident for bracing duration, while differences in extension immobilisation, weight‐bearing progression, and immediate post‐operative ROM were not supported by the available data. Incomplete reporting, particularly for extension‐locking duration, remained a notable limitation for interpretability.

### Overall clinical improvement

**Figure 2:**
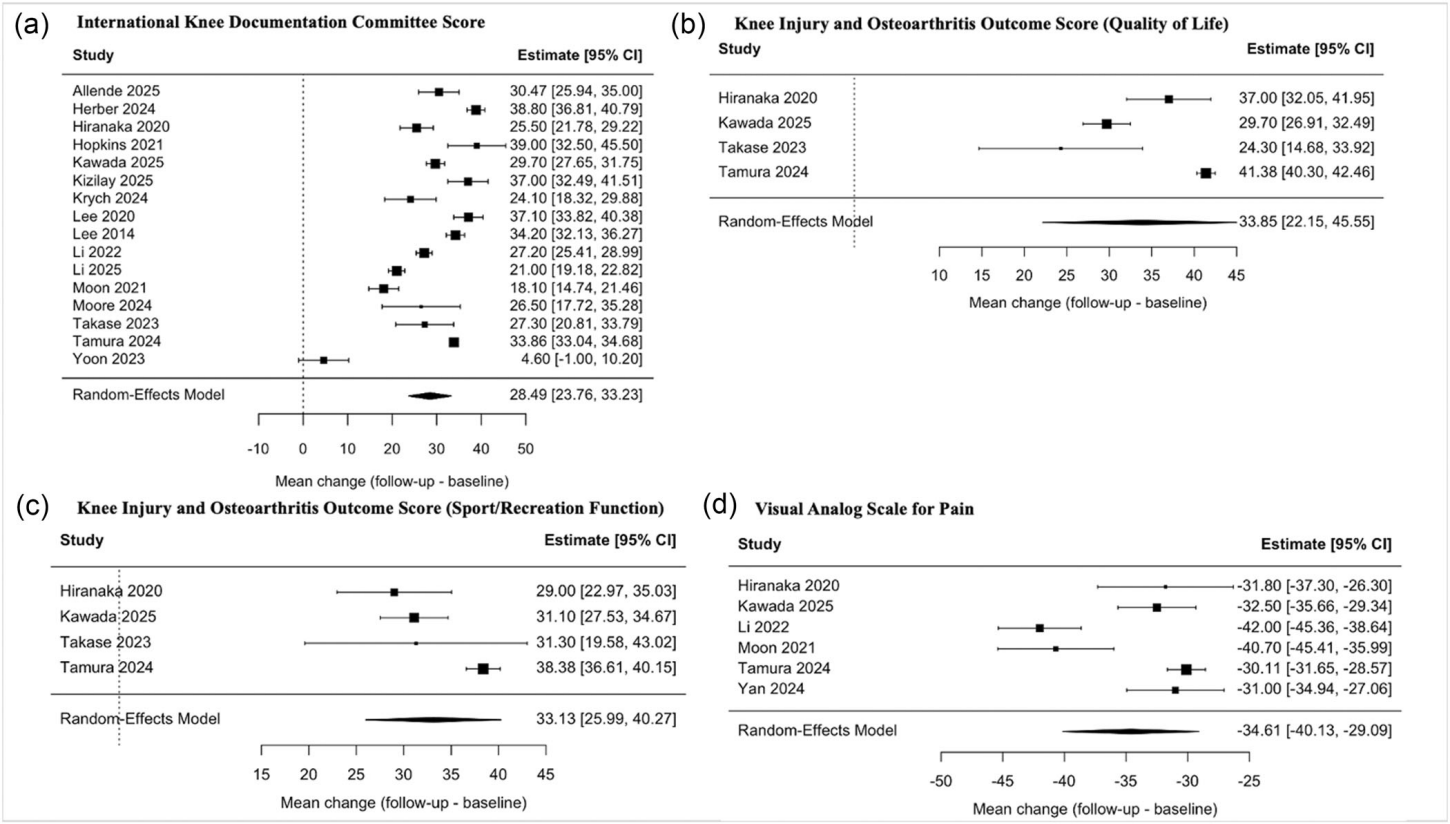
Forest plots of patient‐reported outcomes. (a) International Knee Documentation Committee (IKDC) score. (b) Knee Injury and Osteoarthritis Outcome Score (KOOS) Quality of Life (QoL) subscale. (c) KOOS Sport/Recreation subscale. (d) Visual Analog Scale (VAS) pain score.

Using a correlation coefficient of *r* = 0.5 for change‐from‐baseline calculations, all four PROMs demonstrated statistically significant improvement after transtibial pullout repair of MMPRT (Figure 2). Across 16 studies reporting IKDC with a mean follow up of 2.26 ± 1.71 years, the pooled mean improvement was 28.5 (95% CI: 23.8–33.2; *I* ^2^ = 97.9%). Four studies reported on KOOS subscales QoL and Sport/Recreation, with a mean follow up of 1.72 years for both PROMs (SD not available). The pooled mean change in KOOS QoL was 33.9 (95% CI: 22.2–45.6; *I* ^2^ = 94.8%) and the pooled mean change in KOOS Sport/Recreation was 33.1 (95% CI: 26.0–40.3; *I* ^2^ = 80.5%). Pain, assessed using VAS, was analysed in six studies, with a mean follow up of 1.77 years (SD not available). There were substantial improvements in pain, with pooled mean VAS pain scores decreasing 34.6 points (95% CI: −40.1 to −29.1; *I* ^2^ = 90.0%).

Sensitivity analyses performed with *r* = 0.3 and *r* = 0.7 yielded nearly identical pooled mean changes for all PROMs, with differences limited to approximately ≤0.5 points in either direction (Supporting Information: Appendix 5).

For IKDC outcomes, visual inspection of the funnel plot did not suggest substantial asymmetry (Supporting Information: Appendix 6). Egger's regression test did not detect statistically significant funnel plot asymmetry (*p* = 0.457).

### Post‐operative complications

Post‐operative complications were infrequently reported across the included studies, and definitions, ascertainment windows and denominators varied. Therefore, pooled complication rates could not be reliably estimated. A total of 56 complication events were reported in 13 studies. When reported, complications included repair failure (*n* = 42), infections (*n* = 7), arthrofibrosis requiring procedure (*n* = 2), thromboembolic events (*n* = 1) and reoperation/progression to arthroplasty (*n* = 4).

### Meta‐regression analysis

Meta‐regression was planned for IKDC, KOOS QoL, KOOS Sport/Rec and VAS pain, however only IKDC was reported with sufficient extractable data to permit quantitative synthesis and moderator analyses. Consequently, meta‐regression was restricted to IKDC, and exploratory binary meta‐regression models were used to evaluate whether prespecified rehabilitation protocol variables were associated with between‐study differences in mean IKDC change.

Across all IKDC meta‐regression models, no detectable associations were identified between the evaluated rehabilitation protocol variables and mean IKDC change in the primary analysis (Table 4). Effect estimates were imprecise, with wide confidence intervals spanning the null for brace duration (<6 vs. ≥6 weeks; k = 6 vs. 6, respectively), post‐operative extension locking (yes vs. no; *k* = 7 vs. 8, respectively), immediate post‐operative weight‐bearing status (non‐weightbearing vs. partial weight‐bearing; *k* = 11 vs. 5, respectively), time to weight‐bearing as tolerated (≤ 6 vs. >6 weeks; *k* = 11 vs. 3, respectively), and immediate post‐operative ROM (yes vs. no; *k* = 8 vs. 8), with all *p*‐values ≥ 0.50. Heterogeneity remained substantial in all models (*I* ^2^ = 97.71%–98.15%), indicating that most between‐study variability in IKDC change was not captured by these broad study‐level rehabilitation protocol moderators.

**Table 4:** Meta‐regression analysis for rehabilitation protocol variables and IKDC scores.

Moderator	Reference mean change (*k*)	Alternate mean change (*k*)	*β* (95% CI)	*p*‐value	*I* ^2^ (%)
Brace use duration (<6 [reference] vs. ≥6 weeks)	27.09 (6)	26.27 (6)	−0.67 (−11.81, 10.47)	0.91	97.91
Locked in extension (N [reference] vs. Y)	28.54 (7)	27.13 (8)	−1.47 (−10.76, 7.82)	0.76	97.78
Locked in extension duration^a^ (<2 [reference] vs. ≥2 weeks)	‐	‐	‐	‐	‐
Immediate post‐operative weight‐bearing status (NWB [reference] vs. PWB)	29.17 (11)	26.70 (5)	−2.29 (−11.87, 7.28)	0.64	97.88
Time to weight‐bearing as tolerated (≤6 [reference] vs. >6 weeks)	27.88 (11)	27.18 (3)	0.09 (−11.53, 11.72)	0.99	98.15
ROM allowed immediately postoperatively (N [reference] vs. Y)	27.17 (8)	29.59 (8)	2.44 (−6.47, 11.36)	0.59	97.71

Abbreviations: CI, confidence interval; IKDC, International Knee Documentation Committee; NWB, non‐weightbearing; PWB, partial weight‐bearing; ROM, range of motion.

a IKDC was not reported by enough studies to permit meta‐analysis of this rehabilitation protocol variable.

Sensitivity analyses performed with *r* = 0.3 and *r* = 0.7 yielded nearly identical mean changes for all variables (Supporting Information: Appendices 7a and 7b). Furthermore, sensitivity analyses using alternative dichotomisation thresholds were broadly consistent with the primary meta‐regression findings (Supporting Information: Appendix 8). Alternative thresholds for brace duration and time to weight‐bearing as tolerated did not identify detectable associations with IKDC change, with wide confidence intervals and substantial residual heterogeneity. The only statistically significant alternative‐threshold finding was for locked‐in‐extension duration, where ≥3 weeks was associated with lower IKDC improvement compared with <3 weeks (*β* = −15.4, 95% CI −27.6 to −3.1, *p* = 0.010). However, this analysis included only seven studies and retained very high heterogeneity (*I* ^2^ = 97.7%), so it should be interpreted as exploratory and hypothesis‐generating rather than confirmatory.

## DISCUSSION

The primary findings of this review show that early post‐operative rehabilitation protocols after transtibial pullout repair for MMPRTs are highly variable, while included cohorts generally demonstrated substantial longitudinal improvements in PROMs. Pooled changes in IKDC, KOOS QoL, KOOS Sport/Recreation and VAS pain exceeded commonly cited minimal clinically important difference (MCID) thresholds for meniscal surgery populations, suggesting clinically meaningful average symptomatic and functional improvement after repair [29]. For VAS pain, MCID varies by context and scale anchoring, but commonly cited clinically important changes on a 0–100 mm VAS are on the order of approximately 15–20 mm in musculoskeletal pain settings; [19, 24] the pooled reduction of 34.6 points similarly suggests a clinically meaningful average improvement in pain. The included studies largely used comparator‐free pre‐post designs, so observed improvements cannot be attributed solely to rehabilitation strategy or even to surgery itself; natural history, regression to the mean, placebo/contextual effects, concomitant interventions, and follow‐up duration may also contribute. In addition, heterogeneity was substantial across all PROMs, indicating that pooled values should be viewed as population‐level longitudinal summaries rather than precise expected treatment effects for individual patients.

A central limitation of PROM‐based inference after MMPRT repair is the potential dissociation between symptoms and structural healing. Patients may report improved pain and function despite persistent or progressive meniscal extrusion, incomplete healing, or ongoing cartilage degeneration. Rehabilitation may plausibly influence structural endpoints, including extrusion progression, repair integrity, cartilage preservation and conversion to arthroplasty, differently than it influences short‐ or mid‐term symptoms. Therefore, PROM‐only analyses provide an incomplete basis for determining optimal rehabilitation after MMPRT repair. This is especially relevant because contemporary literature emphasises that root tears alter tibiofemoral contact mechanics and that post‐operative extrusion and alignment may influence longer‐term failure risk. Future studies should pair rehabilitation protocols with imaging, second‐look arthroscopy when available, or longer‐term structural endpoints.

Biomechanical considerations provide a rationale for early protection after MMPRT repair. Meniscal root tears disrupt hoop stress transmission and increase tibiofemoral contact pressures in a manner biomechanically comparable to meniscectomy, while root repair aims to restore more normal contact mechanics [38]. Axial loading and deep flexion may increase stress at the posterior horn, supporting temporary restrictions in weight‐bearing and flexion during early healing. Suture configuration, fixation method, and adjunctive centralisation may also influence construct ability and extrusion control [38]. However, these biomechanical principles should not be over‐translated into clinical certainty as the available clinical literature does not yet define the optimal duration or intensity of post‐operative restrictions after transtibial pullout repair.

Biological factors may also explain why broad rehabilitation variables were unable to account for PROM heterogeneity. MMPRT repair depends on healing at a meniscus‐to‐bone interface in a patient population that often has degenerative tissue quality, meniscal extrusion, varus alignment, elevated BMI, and early cartilage disease. These factors may dominate both healing biology and symptom trajectory. Biologic augmentation strategies, including marrow stimulation, platelet‐rich plasma, fibrin clot, mesenchymal cell‐based approaches, or scaffold/wrapping techniques, have been proposed to improve meniscal healing, but clinical evidence remains limited and heterogeneous [38]. Therefore, symptom improvement should not be assumed to indicate biological healing, and future studies should evaluate rehabilitation protocols alongside structural healing outcomes.

There was substantial heterogeneity in post‐operative rehabilitation practices following transtibial pullout repair of MMPRTs, which should be interpreted in the context of recent ESSKA‐AOSSM‐AASPT consensus guidance [36, 37]. The consensus recommends that rehabilitation after meniscal repair progress according to both time‐ and criterion‐based milestones, with protocol selection influenced by tear pattern, repair stability, tissue quality, and patient‐specific factors. For root repairs specifically, the consensus supports a more protective early approach, including no weight‐bearing for approximately six weeks and ROM limited to 0°–90° for the early post‐operative period [36, 37]. In this review, many protocols broadly aligned with these protective principles, but reporting was too inconsistent and study designs too heterogeneous to determine whether stricter or more accelerated protocols produce superior clinical or structural outcomes. The wide variation in brace duration, weight‐bearing progression, and range‐of‐motion restrictions suggests that post‐operative management is largely driven by surgeon preference, institutional norms, or regional practice patterns rather than comparative outcome data. Regional comparisons remained hypothesis‐generating given the small number of reporting cohorts and absence of adjustment for multiple comparisons. However, bracing duration differed significantly between USA and Asian cohorts, with USA studies more commonly using bracing for ≥6 weeks. In contrast, extension immobilisation, weight‐bearing progression, and immediate ROM did not differ significantly based on available reporting. While not definitive, this pattern suggests differing regional philosophies regarding early mobilisation and thresholds for post‐operative protection, particularly in the setting of concern for early repair‐site strain where deep flexion and axial loading may increase strain at the posterior root [21, 30].

The exploratory study‐level meta‐regression did not identify detectable associations between evaluated early rehabilitation variables and IKDC change. This should not be interpreted as evidence that rehabilitation parameters are unimportant. The analyses were underpowered, used study‐level rather than patient‐level data, and relied on coarse binary categorisations that may obscure dose‐response effects and within‐protocol nuance. High residual heterogeneity suggests that unmeasured clinical and methodological factors such as baseline cartilage status, meniscal extrusion, alignment, chronicity, BMI, surgical technique, centralisation, biologic augmentation, rehabilitation adherence and follow‐up timing likely contributed more to between‐study differences than could be captured by the available rehabilitation variables.

This review contributes a structured synthesis of reported early rehabilitation protocols after transtibial pullout repair for MMPRTs and links these protocols to available PROM data. The large cumulative sample size, inclusion of contemporary studies, and broad geographic representation enhance the generalisability of the findings. Additionally, the consistency of pooled effect estimates across multiple sensitivity analyses supports the robustness of the results despite heterogeneity in study design and rehabilitation practices. However, the value of this review is primarily descriptive and hypothesis‐generating. The findings should not be interpreted as establishing equivalence among rehabilitation protocols, because the evidence base consists predominantly of observational cohorts with variable patient selection, surgical technique, rehabilitation reporting, and follow‐up duration. Overall confidence was highest for the descriptive finding that rehabilitation protocols were heterogeneous, as this was based on direct protocol extraction across 31 studies. Confidence was low for longitudinal PROM improvement and very low for rehabilitation‐moderator inference, reflecting predominantly observational pre‐post designs, limited randomised evidence, substantial clinical/statistical heterogeneity, sparse moderator data, and study‐level ecological bias.

Several limitations should be acknowledged when translating these results into practice. Most included studies were observational cohorts and randomised comparisons of rehabilitation strategies were not available. Pooled PROM improvements were based on pre‐post change scores without nonoperative, surgical, or rehabilitation comparator groups. Rehabilitation reporting was incomplete for several variables, and protocol adherence was generally not reported. Meta‐regression was performed at the study level and is therefore vulnerable to ecological bias, residual confounding, and low power. Dichotomising rehabilitation parameters may obscure dose‐response relationships, and follow‐up duration varied across studies. Clinical heterogeneity was substantial, including differences in age, chronicity, cartilage degeneration, alignment, meniscal extrusion, surgical technique, fixation construct, centralisation and biologic or structural adjuncts. Finally, PROMs alone may not capture structural healing, extrusion progression, cartilage preservation, or longer‐term conversion to arthroplasty.

Future research should prioritise prospective comparative designs that isolate individual rehabilitation components and incorporate both PROMs and structural endpoints (e.g., extrusion progression, healing status, cartilage changes). Given the persistent unexplained heterogeneity in PROM improvements, stratified analyses by baseline degeneration, alignment, extrusion severity, and patient factors (e.g., age, BMI and activity level) will be essential to refining evidence‐based post‐operative care after meniscal root repair.

## CONCLUSIONS

Post‐operative rehabilitation protocols after transtibial pullout repair for MMPRTs are highly variable, particularly for bracing, weight‐bearing progression and ROM restrictions. Across included studies, patients demonstrated substantial longitudinal improvements in IKDC, KOOS QoL, KOOS Sport/Recreation and VAS pain. However, these pre‐post changes should not be interpreted as treatment effects attributable to specific rehabilitation strategies. Exploratory study‐level meta‐regression did not identify detectable associations between broad early rehabilitation parameters and IKDC improvement, but inference is limited by observational designs, high heterogeneity, sparse moderator data, ecological bias and insufficient power.

## AUTHOR CONTRIBUTIONS

All authors contributed to study design, data collection, analysis and manuscript preparation.

## ACKNOWLEDGEMENTS

The authors have no funding to report.

## CONFLICT OF INTEREST STATEMENT

Dr. de SA has the following disclosures, none of which are related to this publication. He is a board member of the Heron Therapeutics Advisory Board and has served as a consultant for L.E.K. Consulting, Atheneum Partners, and Stryker. Additionally, he has participated in the Speakers Bureau for ConMed Linvatec and is a member of the Pendopharm Regional Working Group. The remaining authors declare no competing financial or non‐financial interests related to this work.

## ETHICS STATEMENT

Please include the name of the institutional review board (IRB) and the approval number. If not applicable, please state so.

## Supporting information

Supporting File 1.

Supporting File 2.

## Data Availability

The data that supports the findings of this study are available in the main text of this article.
