# Supplementary material for: Postoperative rehabilitation after transtibial pullout repair of medial meniscus posterior root tears: A systematic review and meta‐analysis
Source: Knee Surg Sports Traumatol Arthrosc. 2026 Jun 16;34(7):2647–60. doi: 10.1002/ksa.70491 (PMC13327495; doi:10.1002/ksa.70491)
Supplement: Supplementary file 1 — Supporting File 1. [file KSA-34-2647-s001.docx]

**Supplementary Appendix 1). MeSH terms and Boolean operators used to develop search strategy from Embase, Medline, SPORTDiscus, and Cochrane Central Register of Controlled Trials**

**Database: Embase <1974 to 2025 June 2>**
**Search Strategy:**

1. (menisc* or meniscal) adj5 root).mp.
2. (posterior adj3 horn adj5 menisc*).mp.
3. (posterior adj3 root adjs menisc*).mp.
4. (root or posteromedial or posterolateral) adjs (tear* or injur* or avulsion* or lesion*) adjs menisc*).mp.
5. (MMPRT or LMPRT).mp.
6. 1 or 2 or 2 or 4 or 5
7. exp surgery/
8. exp arthroscopy/
9. exp orthopedic surgery/
10. exp knee meniscus/
11. (repair* or sutur* or fixat* or reattach* or pull-out* or pullout* or transtibial or tunnel* or anchor* or reconstruction* or centralization*).mp.
12. 7 or 8 or 9 or 10 or 11
13. 6 and 12

**Database: Medline <1946 to June 2, 2025>**
**Search Strategy:**

1. (menisc* or meniscal) adjs root).mp.
2. (posterior adj3 horn adj5 menisc*).mp.
3. (posterior adj3 root) and menisc*).mp.
4. (root or posteromedial or posterolateral) adj5 (tear* or injur* or avulsion* or lesion*) adj5 menisc*).mp.
5. (MMPRT or LMPRT or MMPHT or LMPHT).mp.
6. exp Surgical Procedures, Operative/
7. exp Arthroscopy/
8. exp Orthopedic Procedures/
9. exp Menisci, Tibial/
10. (repair* or sutur* or fixat* or reattach* or pull-out* or pullout* or transtibial or tunnel* or anchor* or reconstruction* or centralization*).mp.
11. 1 or 2 or 2 or 4 or 5
12. 6 or 7 or 8 or 9 or 10
13. 11 and 12

**Database: CINAHL <1986 to 2025>**
**Search Strategy:**

1. TI menisc* N5 root* OR AB menisc* N5 root* OR TI root* N5 menisc* OR AB root* N5 menisc* OR TI TI posterior N3 horn* N5 menisc* OR AB TI posterior N3 horn* N5 menisc* OR TI ( ( (medial OR lateral) N3 menisc* N3 (root OR posterior N1 horn) ) ) OR AB ( ( (medial OR lateral) N3 menisc* N3 (root OR posterior N1 horn) ) ) OR TI ( (posteromedial OR posterolateral OR root*) N5 (tear* OR injur* OR avulsion* OR lesion*) N5 menisc* ) OR AB ( (posteromedial OR posterolateral OR root*) N5 (tear* OR injur* OR avulsion* OR lesion*) N5 menisc* ) OR TI ( (root* OR posterior N1 horn) N5 (repair* OR fixat* OR reattach* OR sutur* OR pullout OR transtibial) ) OR AB ( (root* OR posterior N1 horn) N5 (repair* OR fixat* OR reattach* OR sutur* OR pullout OR transtibial) )
2. TI posterior N3 root* N5 menisc* OR AB posterior N3 root* N5 menisc* OR TI ( MMPRT OR LMPRT OR MMPHT OR LMPHT ) OR AB ( MMPRT OR LMPRT OR MMPHT OR LMPHT )
3. 1 or 2

**Database: SPORTDiscus <1986 to 2025>**
**Search Strategy:**

1. TI menisc* N5 root* OR AB menisc* N5 root* OR TI root* N5 menisc* OR AB root* N5 menisc* OR TI posterior N3 horn* N5 menisc* OR AB posterior N3 horn* N5 menisc* OR TI ( ( (medial OR lateral) N3 menisc* N3 (root OR posterior N1 horn) ) ) OR AB ( ( (medial OR lateral) N3 menisc* N3 (root OR posterior N1 horn) ) ) OR TI ( (posteromedial OR posterolateral OR root*) N5 (tear* OR injur* OR avulsion* OR lesion*) N5 menisc* ) OR AB ( (posteromedial OR posterolateral OR root*) N5 (tear* OR injur* OR avulsion* OR lesion*) N5 menisc* ) OR TI ( (root* OR posterior N1 horn) N5 (repair* OR fixat* OR reattach* OR sutur* OR pullout OR transtibial) ) OR AB ( (root* OR posterior N1 horn) N5 (repair* OR fixat* OR reattach* OR sutur* OR pullout OR transtibial) )
2. TI posterior N3 root* N5 menisc* OR AB posterior N3 root* N5 menisc* OR TI ( MMPRT OR LMPRT OR MMPHT OR LMPHT ) OR AB ( MMPRT OR LMPRT OR MMPHT OR LMPHT )
3. 1 or 2

**Database: EBM Reviews - Cochrane Central Register of Controlled Trials<June 2025>**
**Search Strategy:**

1. ((menisc* or meniscal) adj5 root).mp. [mp=ti, ot, ab, tx, kw, ct, sh, fx, hw]
2. (posterior adj3 horn adj5 menisc*).mp. [mp=ti, ot, ab, tx, kw, ct, sh, fx, hw]
3. ((posterior adj3 root) and menisc*).mp. [mp=ti, ot, ab, tx, kw, ct, sh, fx, hw]
4. ((root or posteromedial or posterolateral) adj5 (tear* or injur* or avulsion* or lesion*) adj5 menisc*).mp. [mp=ti, ot, ab, tx, kw, ct, sh, fx, hw]
5. (MMPRT or LMPRT).mp. [mp=ti, ot, ab, tx, kw, ct, sh, fx, hw]
6. 1 or 2 or 3 or 4 or5
7. (epair* or sutur* or fixat* or reattach* or pull-out* or pullout* or transtibial or tunnel* or anchor* or reconstruction* or centralization*).mp. [mp=ti, ot, ab, tx, kw, ct, sh, fx, hw]
8. 6 and 7

**Supplementary Appendix 2). Additional demographic data of included studies.**

| **Study** | **Time from symptom onset/injury to surgery (months)** | **Meniscal extrusion**  **Pre** → **post (mm)** | **Osteoarthritis/cartilage status** | **Lower limb alignment** | **Repair construct** |
| --- | --- | --- | --- | --- | --- |
| Allende, 2025 | NR | 3.1 ± 1.1 → NR | KL 0/1/2/3/4: 0/27/37/6/0 | FTA 183.2 ± 2.8° | 1–2 transtibial tunnels; 2–3 nonabsorbable sutures; anchor/button fixation |
| Bagherifard, 2025 | 14.4 ± 14.6 | 5.7 ± 1.8 → 3.4 ± 2.5 | NR | NR | One tunnel/socket; two No. 2-0 FiberWire simple cinch sutures; knotless anchor fixation |
| Chung, 2025 | 1.3 ± 0.9 | NR | KL 0/1/2/3/4: 7/20/7/0/0; Outerbridge 0/1/2/3/4: 9/6/12/5/2 | Varus mechanical axis 3.1 ± 2.0° | One tunnel; modified Mason–Allen locking stitch; No. 1 PDS; EndoButton fixation |
| Kawada, 2025 | 2.2 ± 1.8 | 4.3 ± 1.1 → 5.6 ± 1.6 | KL 0/1/2/3/4: 0/75/104/0/0 | FTA 178.0 ± 1.6° | One tibial foramen; two simple or cinch stitches; ± posterior anchoring; screw/anchor fixation |
| Kizilay, 2025 | NR | NR | KL 1/2: 5/36 | NR | One 4.5-mm tunnel; two simple cinch-loop high-strength sutures; EndoButton or screw fixation; no centralization |
| Li, 2025 | NR | NR | NR | NR | Tibial tunnel; nonabsorbable root sutures; cortical button/anchor fixation; configuration NR |
| Nistor, 2025 | 7.0 ± 7.0 | 3.3 ± 1.8 → 3.9 ± 1.7 | Outerbridge 0/1/2/3/4: 1/2/3/4/0 | NR | Two tunnels; 1–2 lasso No. 2 FiberWire sutures; cortical bone-bridge fixation; no centralization reported |
| Tollefson, 2025 | NR | 3.3 ± 0.9 → 5.0 ± 1.3 | KL 1.2 ± 0.6; Outerbridge 2.2 ± 0.8 | HKA 2.3 ± 1.3° | Two transtibial tunnels; vertical mattress suture tape; cortical button fixation |
| Herber, 2024 | NR | 2.8 ± NR → NR | KL 1: 43/97; KL 2: 49/97; other/unclear: 5/97; mean Outerbridge medial/lateral/PF: 1.9/1.0/2.2 | NR | One socket/tunnel; two 0-FiberLink luggage-tag sutures; SwiveLock fixation |
| Krych, 2024 | 4.4 ± 2.9 | 3.2 ± 0.3 → NR | KL 1.3 ± 0.5; Outerbridge 0/1/2/3/4: 0/5/7/12/1 | Varus alignment 2.9 ± 0.9° | One root tunnel/socket plus centralization anchors; 2 cinch sutures + leader stitch; SwiveLock fixation |
| Moore, 2024 | 3.7 ± 3.6 | NR | NR | NR | One tunnel; two high-strength braided locking cinch sutures; cortical button fixation |
| Tamura, 2024 | 2.2 ± 1.8 | 2.1 ± 1.0 → 3.2 ± 1.3 | NR | FTA 177.3 ± 1.4° | One tunnel; two simple stitches plus posteromedial pullout augmentation; interference screw/anchor screw fixation; no centralization |
| Yan, 2024 | NR | 3.7 ± 0.8 → 2.2 ± 0.6 | NR | NR | One 4.5-mm root tunnel; two high-strength root sutures; anchor fixation; centralization anchor used |
| Zhou, 2024 | NR | 4.6 ± 0.9 → 3.3 ± 0.9 | KL 0–1: 72/87; KL 2–4: 15/87 | HKA 2.3 ± 1.1° | One 4.5-mm tunnel; two PDS/high-strength sutures; Endobutton fixation; modified arm added centralization anchor |
| Chen, 2023 | NR | NR | KL 0/1/2/3/4: 2/9/22/14/1 | NR | One tunnel; No. 0 FiberWire; two loop stitches; SwiveLock fixation |
| Takase, 2023 | NR | 3.6 ± 0.9 → 4.3 ± 0.6 | NR | %MA 45.6 ± 3.8; FTA 177.0 ± 1.5° | One tunnel; two cinch-loop sutures; Ultratape/No. 2 FiberWire; double-spike plate/screw fixation; no centralization |
| Yoon, 2023 | NR | 14.2 ± 2.1 → 14.3 ± 1.5 | NR | NR | One tunnel; two simple vertical No. 1 PDS sutures; button fixation |
| Dzidzishvili, 2022 | 8.1 ± NR | NR | KL 1/2/3: 5/19/6; Outerbridge 1/2/3/4: 4/18/4/4 | NR | Transtibial pullout repair; suture, tunnel, and fixation details NR |
| Li, 2022 | NR | NR | KL 0/I/II/III/IV: 14/19/2/0/0 | NR | One tunnel; No. 0 FiberWire root sutures; cortical button fixation |
| Tahami, 2022 | 5.2 ± 2.9 | NR | NR | NR | One tunnel; Loop-Post FiberWire/ExpressBraid construct; screw-washer fixation; no centralization |
| Hopkins, 2021 | 3 ± 3.3 | NR | KL 0/1/2/3/4: 3/13/9/6/0; Outerbridge 0/1/2/3/4: 0/0/9/19/5 | NR | One tunnel; 3 sutures deployed using FAST-FIX 360; EndoButton or suture-anchor fixation |
| Moon, 2021 | 4.4 ± 3.8 | 3.4 ± 0.9 → 4.4 ± 1.3 | KL 0/1/2/3: 18/51/4/0 | HKA 3.2 ± 2.8° varus | One tunnel; modified reverse Mason–Allen; TigerWire + No. 1 PDS; EndoButton fixation |
| Bernard, 2020 | NR | NR | KL 1.6 ± NR | NR | One socket/tunnel; three 0-FiberLink simple cinch/luggage-tag sutures; SwiveLock fixation |
| Hiranaka, 2020 | 2.8 ± 2.1 | NR | NR | NR | Transtibial pullout; modified Mason–Allen or simple-stitch techniques; No. 2 Ultrabraid ± FasT-Fix; screw/plate fixation; tunnel number NR |
| Lee, 2020 | NR | 3.2 ± 0.8 → 3.1 ± 0.9 | KL 1/2/3/4: 6/41/0/0; medial Outerbridge 1/2/3/4: 3/12/27/5 | HKA 2.3 ± 1.0° | One tunnel; modified Mason–Allen No. 1 PDS; EndoButton fixation; atelocollagen/fibrin adjunct in 25/47 |
| Ulku, 2020 | 5.0 ± NR | 3.5 ± 0.4 → 2.5 ± 0.6 | Outerbridge 0/1/2: 16/20/5 | NR | Two tunnels; two loop or simple No. 0 FiberWire sutures; metal button fixation; fibrin clot; no centralization |
| Yanagisawa, 2020 | NR | 3.9 ± 1.0 → 5.1 ± 4.7 | KL 0/1/2: 13/17/0 | FTA 177.8 ± 2.1° | One tunnel; 1–2 cinch-loop 2-0 FiberWire sutures; double-spike plate fixation; no centralization |
| Brophy, 2019 | NR | NR | NR | NR | One tunnel; locking-loop 2-0 FiberWire; poly button fixation |
| Cho, 2014 | 3.1 ± NR | 2.2 ± NR → NR | NR | WBL 40.0% of tibial width | One 6-mm tunnel; two No. 0 PDS pullout strands; screw/washer fixation |
| Lee, 2014 | NR | NR | KL 1/2/3/4: 18/28/4/0 | Mechanical axis 2.5 ± 2.2° varus | One tunnel; modified Mason–Allen or simple vertical No. 1 PDS sutures; button fixation |
| Seo, 2011 | 6.3 ± NR | NR | KL 0/I/II/III: 1/7/2/1; Outerbridge 0/I/II/III: 1/5/2/3 | Varus 3.2 ± 3.3° | One tunnel; two No. 1 PDS sutures; screw/washer fixation |

Abbreviations: FTA: femorotibial angle; HKA: hip-knee-ankle angle; %MA: percentage mechanical axis, describes the mechanical axis location across the tibial plateau as a percentage of tibial width; WBL: weight-bearing line; PDS: polydioxanone suture; HTO: high tibial osteotomy; KL: Kellgren-Lawrence; PF: patellofemoral; NR: not reported.

Values are presented as mean ± SD unless otherwise stated.

**Supplementary Appendix 3). Risk of Bias Assessment using MINORS tool**

| **Study** | **Clearly stated aim** | **Inclusion of consecutive patients** | **Prospective data collection** | **Endpoints appropriate to study aim** | **Unbiased assessment of study endpoint** | **Follow-up period appropriate to study aim** | **Loss to follow-up less than 5%** | **Prospective calculation of study size** | **Adequate control group** | **Contemporary groups** | **Baseline equivalence of groups** | **Adequate statistical analysis** | **Total (/24)** |
| --- | --- | --- | --- | --- | --- | --- | --- | --- | --- | --- | --- | --- | --- |
| Allende, 2025 | 2 | 1 | 1 | 2 | 1 | 2 | 0 | 0 |  |  |  |  | 9* |
| Bagherifard, 2025 | 2 | 1 | 1 | 2 | 1 | 1 | 0 | 0 |  |  |  |  | 8* |
| Chung, 2025 | 2 | 1 | 0 | 1 | 0 | 2 | 0 | 0 | 2 | 0 | 1 | 1 | 10 |
| Kizilay, 2025 | 2 | 1 | 0 | 2 | 1 | 2 | 0 | 0 | 2 | 2 | 1 | 1 | 12 |
| Nistor, 2025 | 2 | 1 | 0 | 2 | 1 | 2 | 1 | 1 |  |  |  |  | 10* |
| Kawada, 2025 | 2 | 1 | 0 | 2 | 1 | 2 | 1 | 1 |  |  |  |  | 10* |
| Herber, 2024 | 2 | 2 | 1 | 2 | 1 | 2 | 1 | 0 | 2 | 2 | 1 | 1 | 17 |
| Krych, 2024 | 2 | 1 | 1 | 2 | 1 | 1 | 1 | 0 |  |  |  |  | 9* |
| Moore, 2024 | 2 | 1 | 0 | 2 | 1 | 2 | 1 | 1 |  |  |  |  | 10* |
| Tamura, 2024 | 2 | 1 | 0 | 2 | 1 | 2 | 1 | 0 | 1 | 0 | 2 | 2 | 14 |
| Yan, 2024 | 2 | 1 | 0 | 2 | 1 | 2 | 0 | 1 |  |  |  |  | 9* |
| Zhou, 2024 | 2 | 1 | 0 | 2 | 0 | 2 | 2 | 0 | 2 | 2 | 2 | 1 | 16 |
| Chen, 2023 | 2 | 1 | 1 | 2 | 0 | 2 | 1 | 1 |  |  |  |  | 10* |
| Takase, 2023 | 2 | 1 | 2 | 2 | 2 | 2 | 2 | 2 |  |  |  |  | 15* |
| Yoon, 2023 | 2 | 2 | 0 | 1 | 1 | 2 | 1 | 1 | 2 | 1 | 1 | 1 | 15 |
| Dzidzishvili, 2022 | 2 | 1 | 0 | 2 | 0 | 2 | 1 | 1 |  |  |  |  | 9* |
| Li, 2022 | 2 | 1 | 1 | 2 | 2 | 2 | 1 | 0 | 2 | 2 | 2 | 1 | 18 |
| Tahami, 2022 | 2 | 2 | 0 | 2 | 0 | 2 | 0 | 0 | 2 | 0 | 0 | 1 | 11 |
| Hopkins, 2021 | 2 | 1 | 1 | 2 | 1 | 2 | 1 | 0 |  |  |  |  | 10* |
| Moon, 2021 | 2 | 1 | 0 | 2 | 2 | 2 | 0 | 2 | 2 | 2 | 1 | 2 | 18 |
| Bernard, 2020 | 2 | 1 | 1 | 2 | 1 | 2 | 1 | 1 |  |  |  |  | 11* |
| Hiranaka, 2020 | 2 | 2 | 0 | 2 | 1 | 2 | 2 | 0 | 2 | 1 | 2 | 2 | 18 |
| Lee, 2020 | 2 | 1 | 0 | 2 | 1 | 2 | 1 | 1 | 2 | 2 | 2 | 1 | 17 |
| Ulku, 2020 | 2 | 1 | 1 | 2 | 1 | 2 | 1 | 1 | 2 | 2 | 1 | 1 | 17 |
| Yanagisawa, 2020 | 2 | 2 | 0 | 2 | 1 | 2 | 0 | 0 | 0 | 2 | 0 | 1 | 12 |
| Brophy, 2019 | 2 | 1 | 1 | 2 | 1 | 1 | 1 | 0 |  |  |  |  | 9* |
| Cho, 2014 | 2 | 2 | 0 | 2 | 1 | 2 | 2 | 0 | 1 | 1 | 1 | 1 | 15 |
| Lee, 2014 | 2 | 1 | 0 | 2 | 1 | 1 | 0 | 1 | 2 | 0 | 2 | 1 | 13 |
| Seo, 2011 | 2 | 1 | 0 | 2 | 1 | 1 | 1 | 0 |  |  |  |  | 8* |

*This study was a non-comparative study, and so was scored out of 16 based on the first eight criteria only.

**Supplementary Appendix 4). Risk of Bias Assessment using Cochrane Risk of Bias 2.0 (RoB 2) tool**

| **Study** | **Domain 1** | **Domain 2** | **Domain 3** | **Domain 4** | **Domain 5** | **Overall** |
| --- | --- | --- | --- | --- | --- | --- |
| Li, 2025 | Some concerns | Some concerns | Some concerns | High risk | Some concerns | High risk |
| Tollefson, 2025 | Low risk | Some concerns | Some concerns | Some concerns | Some concerns | Some concerns |

**Supplementary Appendix 5). Overall pooled mean change scores with r = 0.5, and sensitivity analyses with r = 0.3 and r = 0.7**

| **PROM** | **r** | **k** | **Pooled mean change** | **95% CI low** | **95% CI high** | **τ²** | **I²** |
| --- | --- | --- | --- | --- | --- | --- | --- |
| IKDC | 0.3 | 16 | 28.51 | 23.78 | 33.24 | 71.30 | 97.1 |
| KOOS QoL | 0.3 | 4 | 34.08 | 22.50 | 45.66 | 42.94 | 92.5 |
| KOOS Sport/Rec | 0.3 | 4 | 33.32 | 26.10 | 40.54 | 17.54 | 74.5 |
| VAS pain | 0.3 | 6 | -34.56 | -40.08 | -29.05 | 23.04 | 86.6 |
| IKDC | 0.5 | 16 | 28.49 | 23.76 | 33.23 | 73.23 | 97.9 |
| KOOS QoL | 0.5 | 4 | 33.85 | 22.15 | 45.56 | 45.70 | 94.8 |
| KOOS Sport/Rec | 0.5 | 4 | 33.13 | 25.99 | 40.27 | 17.81 | 80.5 |
| VAS pain | 0.5 | 6 | -34.61 | -40.13 | -29.09 | 24.21 | 90.0 |
| IKDC | 0.7 | 16 | 28.47 | 23.73 | 33.22 | 75.18 | 98.7 |
| KOOS QoL | 0.7 | 4 | 33.60 | 21.77 | 45.43 | 49.29 | 97.0 |
| KOOS Sport/Rec | 0.7 | 4 | 32.92 | 25.90 | 39.94 | 17.94 | 87.2 |
| VAS pain | 0.7 | 6 | -34.66 | -40.18 | -29.13 | 25.43 | 93.4 |

Abbreviations: IKDC, International Knee Documentation Committee; KOOS, Knee Injury and Osteoarthritis Outcome Score; QoL, Quality of Life; VAS, Visual Analog Scale; CI, confidence interval.

**Supplementary Appendix 6). Funnel plot for IKDC.**


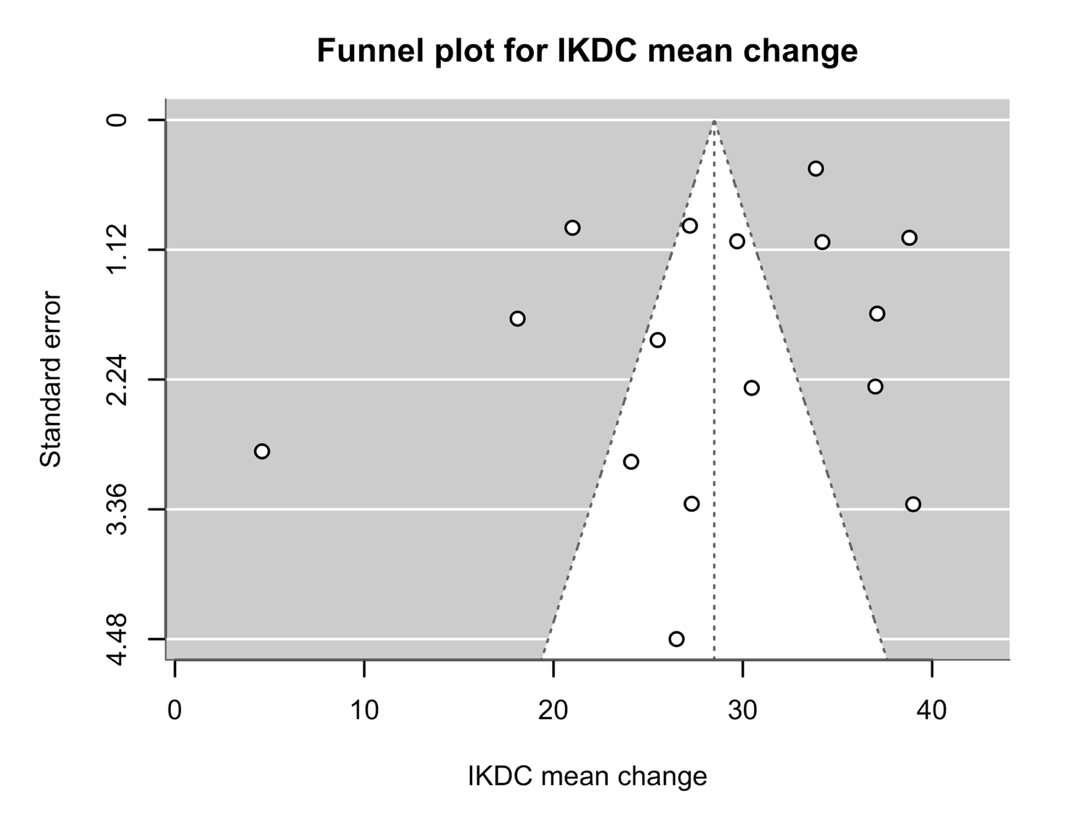


**Supplementary Appendix 7a). Sensitivity analysis for meta-regression of rehabilitation protocol variables and IKDC scores; r = 0.3**

| **Moderator** | **Reference mean change (k)** | **Alternate mean change (k)** | **β (95% CI)** | **P-value** | **I² (%)** |
| --- | --- | --- | --- | --- | --- |
| **Brace use duration**  (<6 [reference] vs ≥6 weeks) | 27.14 (6) | 26.26 (6) | -0.69 (-11.84, 10.45) | 0.90 | 97.08 |
| **Locked in extension**  (N [reference] vs Y) | 28.45 (7) | 27.17 (8) | -1.36 (-10.64, 7.92) | 0.77 | 96.88 |
| **Locked in extension duration***  (<2 [reference] vs ≥2 weeks) |  |  |  |  |  |
| **Initial postoperative weightbearing status**  (NWB [reference] vs PWB) | 29.13 (11) | 26.74 (5) | -2.18 (-11.74, 7.37) | 0.65 | 97.03 |
| **Time to weight-bearing as tolerated**  (≤6 [reference] vs >6 weeks) | 28.14 (3) | 27.22 (11) | 0.11 (-11.56, 11.78) | 0.98 | 97.40 |
| **ROM allowed immediately postoperatively**  (N [reference] vs Y) | 27.21 (8) | 29.52 (8) | 2.33 (-6.58, 11.23) | 0.61 | 96.80 |

*IKDC was not reported by enough studies to permit meta-analysis of this rehabilitation protocol variable

Abbreviations: NWB, non-weight bearing; PWB, partial weight bearing.

**Supplementary Appendix 7b). Sensitivity analysis for meta-regression of rehabilitation protocol variables and IKDC; r = 0.7**

| **Moderator** | **Reference mean change (k)** | **Alternate mean change (k)** | **β (95% CI)** | **P-value** | **I² (%)** |
| --- | --- | --- | --- | --- | --- |
| **Brace use duration**  (<6 [reference] vs ≥6 weeks) | 27.03 (6) | 26.28 (6) | -0.64 (-11.77, 10.50) | 0.91 | 98.73 |
| **Locked in extension**  (N [reference] vs Y) | 28.64 (7) | 27.09 (8) | -1.58 (-10.88, 7.72) | 0.74 | 98.64 |
| **Locked in extension duration***  (<2 [reference] vs ≥2 weeks) |  |  |  |  |  |
| **Immediate postoperative weight-bearing status**  (NWB [reference] vs PWB) | 29.20 (11) | 26.66 (5) | -2.40 (-12.00, 7.20) | 0.62 | 98.71 |
| **Time to weight-bearing as tolerated**  (≤6 [reference] vs >6 weeks) | 27.61 (3) | 27.14 (11) | 0.07 (-11.51, 11.65) | 0.99 | 98.89 |
| **ROM allowed immediately postoperatively**  (N [reference] vs Y) | 27.12 (8) | 29.67 (8) | 2.56 (-6.35, 11.47) | 0.57 | 98.61 |

*IKDC was not reported by enough studies to permit meta-analysis of this rehabilitation protocol variable

Abbreviations: NWB, non-weight bearing; AWB, partial weight bearing.

**Supplementary Appendix 8). Sensitivity analysis for meta-regression of rehabilitation protocol variables and IKDC; alternative thresholds**

| **Moderator** | **Reference mean change (k)** | **Alternate mean change (k)** | **β (95% CI)** | **P-value** | **I² (%)** |
| --- | --- | --- | --- | --- | --- |
| **Brace use duration**  (<5 [reference] vs ≥5 weeks) | 27.09 (6) | 26.27 (6) | -0.67 (-11.81, 10.47) | 0.91 | 97.91 |
| **Brace use duration***  (<7 [reference] vs ≥7 weeks) |  |  |  |  |  |
| **Locked in extension duration***  (<1 [reference] vs ≥1 weeks) |  |  |  |  |  |
| **Locked in extension duration**  (<3 [reference] vs ≥3 weeks) | 34.12 (3) | 18.87 (4) | -15.37 (-27.64, -3.10) | 0.01 | 97.72 |
| **Time to weight-bearing as tolerated**  (≤5 [reference] vs >5 weeks) | 27.88 (3) | 27.18 (11) | 0.09 (-11.53, 11.72) | 0.99 | 98.15 |
| **Time to weight-bearing as tolerated**  (≤7 [reference] vs >7 weeks) | 28.66 (11) | 20.45 (3) | -7.17 (-17.99, 3.66) | 0.19 | 97.77 |

*IKDC was not reported by enough studies to permit meta-analysis of this rehabilitation protocol variable
